# A Communitywide Collaboration to Increase Enrollment, Retention, and Success in Evidence-Based Lifestyle-Change Programs in Racial and Ethnic Minority Populations

**DOI:** 10.5888/pcd20.220352

**Published:** 2023-08-03

**Authors:** Maura Kepper, Katherine A. Stamatakis, Natalie Mudd, Ariel Deitch, Ally Terhaar, Julia Liu, Emerald Gates, Bobie Williams, Gabrielle Cole, Carolyn S. French, Amy Hampton, Amy Eyler

**Affiliations:** 1 Prevention Research Center, Washington University in St. Louis, St. Louis, Missouri; 2 College for Public Health and Social Justice, Washington University in St. Louis, St. Louis, Missouri; 3 St. Louis County Department of Public Health, St. Louis, Missouri; 4 City of St. Louis Department of Health, St. Louis, Missouri; 5 Fit and Food Connection, St. Louis, Missouri; 6 Gateway Region YMCA, St. Louis, Missouri; 7 Missouri Department of Health and Senior Services, Bureau of Cancer and Chronic Disease Prevention, Jefferson City, Missouri

## Abstract

**Purpose and Objectives:**

Chronic diseases (eg, diabetes, hypertension) are the leading causes of death in the US and disproportionally affect racial and ethnic minority populations. This disparity is partially due to the unequal burden of unmet social needs that stem from several factors, including racism.

**Intervention Approach:**

The Alliance is a collaboration among health care, public health, and community organizations formed to improve referral, enrollment, and successful completion of evidence-based lifestyle-change programs, particularly among Black people. The Alliance built 1) a system to assess and address social barriers through the screening and referral process and 2) a training center for frontline staff (eg, community health workers).

**Evaluation Methods:**

From January 2020 through September 2022, we conducted an evaluation that included both quantitative and qualitative methods. We developed an electronic database to make referrals and track key barriers to participation. Additionally, we conducted a focus group among frontline staff (N = 15) to understand the challenges in making referrals and discussing, documenting, and addressing barriers to participation. We used surveys that collected quantitative and open-ended qualitative responses to evaluate the training center and to understand perceptions of training modules as well as the skills gained.

**Results:**

Frontline staff engaged with 6,036 people, of whom 847 (14%) were referred to a lifestyle-change program from January 2020 through September 2022. Of those referred, 257 (30%) were eligible and enrolled in a program. Food access and unreliable internet were the most common barriers to participation. Thirteen of 15 frontline staff participated in trainings, and, on average, trainees completed 4.2 trainings and gained several skills (eg, ability to monitor personal bias, de-escalate a crisis, educate on mental health, understand community and environmental factors).

**Implications for Public Health:**

The Alliance is an example of how health care, public health, and community partners can work together to increase enrollment in lifestyle-change programs of residents disproportionately affected by chronic diseases. Lessons learned from implementation and evaluation can inform other complex partnerships to improve public health.

**Summary**

**What is already known on this topic?**

Evidence-based lifestyle-change programs can reduce the burden of chronic disease. Unmet social needs disproportionately affect Black populations and the ability to enroll in and complete lifestyle-change programs.

**What is added by this report?**

We describe an example of how health care, public health, and community partners can work together to increase recruitment, enrollment, and success of Black people in evidence-based lifestyle-change programs.

**What are the implications for public health practice?**

Lessons learned from implementation and evaluation of lifestyle-change programs may be applied to other complex partnerships between clinical and community-based organizations to improve the health and well-being of people who are disproportionately affected by chronic disease.

## Introduction

Chronic diseases such as diabetes, heart disease, hypertension, and stroke are the leading causes of illness, disability, and death in the US (1). Approximately half of the US population has a chronic disease, and these diseases account for 86% of all health care costs (2,3). More than 133 million Americans have diabetes (37.3 million) or prediabetes (96 million) (4). Diabetes and other chronic diseases disproportionally affect racial and ethnic minority groups. In 2018 in St. Louis City, the disparate burden of diabetes offered a stark example: the prevalence of diabetes was 13.4% among Black residents and 5.5% among non-Hispanic White residents, while diabetes mortality was 26.8 per 100,000 Black residents and 21.0 per 100,000 non-Hispanic White residents (5). Chronic diseases are affected by interdependent genetic, social, economic, cultural, and historical factors (6). The unequal burden of unmet social needs among Black people also contributes to chronic disease disparities (4,7).

The disparity in unmet social needs among Black people stems from racism, the unjust social, economic, and political oppression of non-Hispanic White people in the US. Racism occurs at multiple levels, including systemic racism, which creates structural barriers to health care access, and interpersonal racism, enacted by health care providers on their patients (7,8). Unmet social needs not only affect the risk of developing a chronic disease but also contribute to a disproportionate level of complications among non-Hispanic Black people (9,10). Despite the higher prevalence of chronic diseases and complications among Black people, they are less likely to receive recommended preventive care (9,11). The work described here focuses on addressing interpersonal racism, by training frontline staff who provide care for Black people, and structural racism, by providing resources to address unmet social needs that stem from inequitable environments and systems.

The Centers for Disease Control and Prevention (CDC) developed a suite of evidence-based lifestyle-change programs (LCPs) that provide preventive services through community organizations (eg, the YMCA). The Diabetes Prevention Program (DPP) was established in 2010 and is an evidenced-based LCP designed to prevent or delay the onset of type 2 diabetes (12). The CDC-approved curriculum — written at the 6th-grade reading level — is a year-long program instructed by lifestyle coaches with the goal of helping participants achieve a healthier lifestyle that encompasses nutrition changes, increased physical activity, and stress reduction (12). The DPP has demonstrated that lifestyle changes can be more effective than prescription medication to prevent or delay the onset of type 2 diabetes (13). The DPP Research Group found that 58% of people with prediabetes and 71% of people aged older than 60 years were able to meet the goal of decreasing body weight by 5% to 7% (14). Virtual DPP programs have helped people to meet weight-loss goals, especially people with low incomes and prediabetes who may not be able to attend in-person LCPs (13). The blood pressure self-monitoring program is a 4-month program developed by CDC to help participants measure their blood pressure correctly and consistently and educate them on healthy eating. Self-monitoring of blood pressure is supported by numerous national agencies (eg, American Heart Association) and can improve the management of hypertension (15).

Despite the evidence base for these programs, not everyone has an equal opportunity to access and succeed in these programs. Barriers to enrollment and participation exist, such as poor access to nutritious foods, few safe environments for physical activity, lack of transportation to programs, lack of reliable internet access or technology, and lack of childcare. Such barriers disproportionately affect Black people and families and may contribute to disparities in enrollment, retention, and success in LCPs (16). Screening for social needs allows providers to clearly identify barriers faced by program participants and determine how to effectively intervene. Interventions that alleviate unmet needs through screening, referral, and tracking of patients are imperative to increasing enrollment and success in LCPs (17).

## Purpose and Objectives

The Alliance program was formed across multiple community-based health organizations in the St. Louis metropolitan area to design, test, and evaluate innovations that will optimize health status and advance racial equity. A major focus of the Alliance was to improve the reach of LCPs, particularly among Black residents living in the federally designated Promise Zone. Promise Zones are high-poverty, often medically underserved communities where the federal government partners with local leaders to enhance public health (18). These areas were formed by centuries of racial prejudice that resulted in migration patterns, both voluntary and forced, and territorial acquisition that led to the concentration of racial and ethnic minority groups (19). The largest of 22 Promise Zones in the US, the St. Louis regional Promise Zone comprises 25 zip codes in the northern region of the city and county, an area that is home primarily to Black residents.

The objective of this article is to describe the process and preliminary outcomes of the implementation and evaluation of the Alliance program. It will provide insight and describe lessons learned on addressing interpersonal and structural barriers to improving antiracist efforts in chronic disease prevention and summarize factors that affected the ability of the Alliance to refer and enroll members of a racial minority group, specifically low-income Black people, in LCPs.

## Intervention Approach

The Alliance is a partnership among the Missouri Department of Health and Senior Services, the St. Louis County Department of Public Health, the City of St. Louis Department of Health, the Integrated Health Network, the Missouri Primary Care Association, the Missouri Pharmacy Association, Fit and Food Connection, and the Gateway Region YMCA (Figure). The partnership was funded by CDC’s Division of Diabetes Translation DP18-1817 project, a 5-year cooperative agreement, which launched October 1, 2018, and ends September 30, 2023. The project funds health departments to develop new and innovative approaches to increase the reach and effectiveness of evidence-based public health strategies in populations and communities with a high burden of diabetes, heart disease, and stroke (20).

**Figure:**
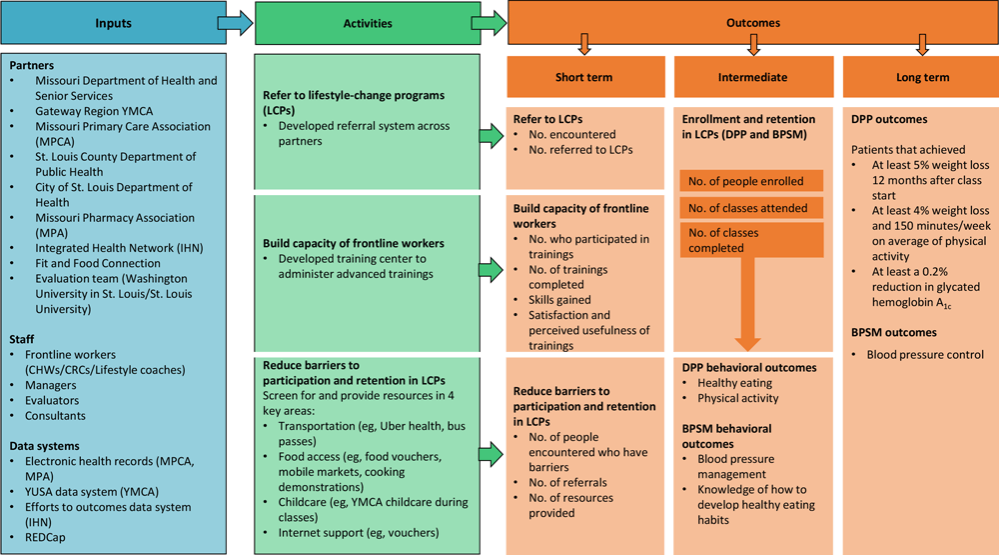
The Alliance logic model. Abbreviations: BPSM, blood pressure self-monitoring; CHW, community health worker; CRC, community resource coordinator; DPP, Diabetes Prevention Program; IHN, Integrated Health Network; LCP, lifestyle change programs; MPA, Missouri Pharmacy Association; MPCA, Missouri Primary Care Association; REDCap, Research Electronic Data Capture; YMCA, Young Men’s Christian Association; YUSA, YMCA of the United States of America.

The main provider of the national DPP program and other LCPs (eg, the blood pressure self-monitoring program) in St. Louis is the Gateway Region YMCA. The Alliance supports community health workers and community resource coordinators, referred to as frontline workers, at partner organizations to screen patients for diabetes and hypertension risk and make referrals to LCPs. Lifestyle coaches, also considered frontline workers, facilitate programs and further support patients once they are enrolled in a program. Lifestyle coaches work with community health workers, community resource coordinators, and a community health navigator, who is embedded in the YMCA, to address social needs throughout the program with the goal of supporting people to complete the 12-month DPP.

### Assessing and addressing social needs

The Alliance program developed a system to identify social barriers that may challenge full participation and success in LCPs. The system allows frontline staff at partner organizations to direct participants to other community programs and resources (eg, food assistance programs) that support health and well-being. For those who enroll in an LCP, the Alliance provides access to food vouchers, YMCA memberships, cooking and wellness-related classes, transportation subsidies, and onsite childcare to improve equity in enrollment, retention, and completion. Community health workers and partner organizations created a list of resources and a process for recommending, using, or accessing these resources to address patient barriers to participation.

### Training center for frontline staff

The Alliance also built the capacity of frontline staff to interact with people disproportionately affected by chronic diseases, specifically Black residents, in community and clinical settings without the intention of inflicting interpersonal racism. To support a well-rounded and versatile workforce and offer high-quality training opportunities, the Alliance launched a training center in year 2.

Participation in training modules was not required of frontline staff but was strongly encouraged. Project staff created an online hub to notify frontline staff of training opportunities. A bootcamp-style training, including an introduction to relevant partners, resources, and procedures, was developed to orient frontline staff to the Alliance project. This training is now required of all new frontline staff and remains available for staff to take multiple times if needed.

## Evaluation Methods

The Alliance used a strategic evaluation planning process for its evaluation. This process facilitates a transparent, logical, and participatory approach for assessing program and project-level outcomes (21). The strategic evaluation planning process involved 2 key groups throughout planning and evaluation: 1) program operators (eg, coalition partners, staff) and 2) primary users of the evaluation (eg, sponsors, collaborators, managers, partners). In year 1 (October 2018–September 2019), the Alliance evaluation team worked collaboratively with each partner to set up equitable data collection and reporting systems tailored to each organization while ensuring the collection of information needed for the overall evaluation. Outcomes were selected to align with 1) the goal of increasing the number of people, especially Black people, referred to, enrolled in, and successful in LCPs and 2) each organization’s reporting systems and capacity to ensure that data collection and reporting were realistic and sustainable.

**Quarterly data report.** The team created a quarterly data report that aggregated information from each partner and communicated progress toward program goals. In this highly collaborative, multi-partner program, consisting of many interrelated strategies, these data reports provided a mechanism for the Alliance leadership to manage risks and challenges that could impede successful implementation. Quarterly data reports were presented in all-partner meetings, distributed by email, and uploaded to a shared drive, which gave partners on-demand access to information on the progress and results of the evaluation project.

**Referral system. **The project team used REDCap (Research Electronic Data Capture) software hosted at Washington University in St. Louis. REDCap is a secure, web-based software platform designed to support data capture for research studies. We developed an electronic form and database in REDCap that launched in January 2020 and allowed all Alliance partners to make referrals to the YMCA through a common pathway. The referral form included information about the frontline staff member making the referral and their Alliance organization to allow for tracking at the organization level and allow the YMCA to communicate with the referring organization about the status of the person referred (eg, whether they enrolled, were actively engaged, or completed the program). The YMCA monitored referrals via REDCap in real time and used the system to track enrollment information and patient demographic data.

**Addressing social needs and averting interpersonal racism.** In addition to the referral system and quarterly data reports, the evaluation team used quantitative and qualitative approaches to examine 2 key strategies used by the Alliance: 1) accounting for social needs and barriers to participation and 2) building the capacity of frontline staff to interact with racial and ethnic minority populations in ways that do not inflict interpersonal racism. The referral system allowed frontline staff to document 4 barriers to participation identified by the Alliance partners as key to enrolling and being successful in LCPs: lack of transportation, food insecurity, lack of reliable internet, and childcare needs. Each organization had its own method for assessing social needs.

**Focus group.** Ten months after launching the referral system, the evaluation team conducted a focus group with frontline staff to understand the challenges of discussing, documenting, and addressing barriers to participation and making referrals to LCPs. The focus group was conducted virtually during the regular bimonthly meeting of frontline staff. Questions were developed to gain insight into the experiences of the frontline staff during their encounters with patients. Questions addressed social barriers that affect patients’ ability to stay healthy, challenges in assessing unmet social needs, resources for patients’ needs, and sustainability of assessing social needs after the Alliance project ends. The session was recorded and transcribed verbatim for analysis. Additionally, interactive all-partner activities were conducted throughout the project to refine processes across organizations. For example, frontline staff and managers from all partner organizations participated in mapping referral pathways and amending language on the referral form to better fit the needs of partners.

**Training center.** To evaluate the training center, project staff monitored participation in each training module and provided participants with a pre- and postsurvey to measure short-term changes in knowledge and frontline staff perception of training module effectiveness. Additionally, annual surveys were distributed to all participants to assess long-term maintenance and application of knowledge and skills. These annual surveys included open-ended questions to allow for qualitative responses. Data quality issues emerged with the pre- and postsurvey collection due to changes in the implementation platform. As a result, presurvey and postsurvey results are not reported. For this evaluation, we have results only for the annual survey conducted in September 2021, during year 3 (October 2020–September 2021). Year 4 (October 2021–September 2022) and year 5 (October 2022–September 2023) annual surveys had not been administered at the time of this writing. Barriers and facilitators of developing and implementing the training center were documented through informal discussions with relevant program staff and managers.

### Evaluation framework

We used the Practical, Robust Implementation, and Sustainability Model (PRISM) to consider the dimensions of reach, effectiveness, adoption, and implementation and how they are influenced by multiple levels (ie, person, intervention, clinic or organization, and environment) (22). Year 1 of the 5-year project was used for hiring, planning, and establishing evaluation processes and systems for engaging the community and making referrals to LCPs. Outcomes for all 5 years of the project were guided by the Reach, Effectiveness, Adoption, Implementation and Maintenance (RE-AIM) outcomes, which are part of the PRISM framework (Table 1). Reach was assessed as the absolute number of people encountered, defined as an interaction between an Alliance frontline staff member and a community member who could benefit from an LCP. A referral is a result of an encounter whereby a connection to LCPs is provided to the participant. The reach of the training center was examined as the number and proportion of frontline staff who participated in trainings. Effectiveness was defined as making referrals and enrolling people, especially those in the Promise Zone, in LCPs, and providing support for unmet social needs. The effectiveness of the training center was assessed as skills gained from trainings. Adoption was operationalized at the organizational level to understand which partners were participating in referrals and trainings. In the future, evaluation data will allow examination of retention and success (eg, improvements in health behaviors and outcomes) of program participants who received referrals (Figure). Additionally, the evaluation team will examine whether people who received the needed social support (through community resources, vouchers, etc) had better participation, retention, and success in the program than people who did not receive such support. As highlighted in PRISM, it was critical to realize the importance of context when examining the implementation of the Alliance project because it aimed to coalesce multiple organizations, each of which had its own resources, systems, cultures, and setting.

**Table 1:** Outcomes Guided by the Reach, Effectiveness, Adoption, Implementation, and Maintenance (RE-AIM) Framework in an Evaluation of a Project to Increase Participation of Black People in Evidence-Based Lifestyle-Change Programs, St. Louis, 2018–2023^a^

RE-AIM construct	Outcomes	Data sources
Reach	The absolute number of community members who were encountered (years 2–4)	Quarterly data reports; REDCap referral system
	The absolute number and proportion of frontline staff who participated in trainings (year 3)	REDCap Training Center survey
Effectiveness	The absolute number, proportion, and representativeness of community members referred and enrolled (years 2–4)	REDCap referral system
	Skills gained from trainings (year 3)	REDCap Training Center survey
Adoption	The absolute number and proportion of Alliance organizations that made referrals and participated in trainings (years 2–4)	REDCap referral system; REDCap Training Center survey
Implementation	Barriers and facilitators to implementing and evaluating the Alliance programs (eg, making referrals, addressing social needs, training frontline staff) (years 1–4)	Process data; focus groups

a The study period was January 2020–September 2022. The project was funded by the Centers for Disease and Control’s Division of Diabetes Translation DP18-1817 project, a 5-year cooperative agreement, which launched October 1, 2018, and ends September 30, 2023.

### Data analysis

We used descriptive statistics and SAS version 9.4 software (SAS Institute Inc) to analyze all quantitative data. A single rater used rapid qualitative analysis methods (23) to analyze qualitative data (focus group, meetings, training center surveys); these methods were validated by other evaluation team members. The qualitative data from the focus group were analyzed by using a priori codes based on the interview guides. Two team members read through and coded the text from the discussion and then talked through discrepancies for reliability. Themes were derived from the coded text and summarized. Thematic summaries were aggregated into a brief and presented to Alliance partners.

## Results

### Referral and enrollment

The Alliance had 15 frontline staff members during the study period (January 2020–September 2022), with an average of 13 per year across partners. These staff members engaged with 6,036 people. Engagement increased as capacity (eg, number of frontline staff members, training, partnerships) increased (Table 2). On average, each frontline staff member engaged 234 people annually. Of the people encountered from January 2020 to September 2022, 847 (14%) were referred to the YMCA for an LCP (approximately 25 referrals per month). All 7 Alliance organizations referred community members to the YMCA. Referred people were aged on average 54.7 years (Table 3). Most (78%) were female and living in the Promise Zone (55%); 21% were food insecure, 15% had transportation needs, 3% needed childcare support, and 30% had unreliable internet.

**Table 2:** Engagement in a Project to Increase Participation of Black People in Evidence-Based Lifestyle-Change Programs, St. Louis, 2018–2023^a^

Phase	Year 2 (October 2018–September 2019)	Year 3 (October 2019–September 2020)	Year 4 (October 2020–September 2021)	Total
Engaged	1,917	1,915	2,204	6,036
Referred	317	230	300	847
Enrolled	50	99	108	257

a The study period was January 2020–September 2022. The project was funded by the Centers for Disease and Control’s Division of Diabetes Translation DP18-1817 project, a 5-year cooperative agreement, which launched October 1, 2018, and ends September 30, 2023.

**Table 3:** Representativeness of Participants in Lifestyle-Change Programs, St. Louis, 2018–2023^a^

Characteristic	Total referred (n = 847)^b^	Total enrolled (n = 257)^b^
**Age**		
Respondents to question	798 (94.2)	257 (100.0)
Mean (SD), y	54.7 (13.2)^c^	55.3 (13.1)^c^
Missing data	49 (5.8)	0
**Sex**		
Respondents to question	837 (98.8)	257 (100.0)
Male	179 (21.4)^c^	20 (7.8)^c^
Female	655 (78.3)^c^	237 (92.2)^c^
Unspecified	3 (0.4)^b^	0
Missing data	10 (1.2)	0
**Reside in the Promise Zone^d^**		
Respondents to question	799 (94.3)	257 (100.0)
Respondents who reside in Promise Zone	440 (55.1)^c^	115 (44.7)
Missing data	48 (5.7)	0
**Social barriers to participation**		
Lack of food access		
Respondents to question	568 (67.1)	228 (88.7)
Respondents with lack of food access	119 (21.0)^c^	33 (14.5)^c^
Missing data	279 (32.9)	29 (11.3)
Transportation needs		
Respondents to question	564 (66.6)	226 (87.9)
Respondents with transportation needs	83 (14.7)^c^	21 (9.3)^c^
Missing data	283 (33.4)	31 (12.1)
Childcare needs		
Respondents to question	564 (66.6)	227 (88.3)
Respondents with childcare needs	16 (2.8)^c^	3 (1.3)^c^
Missing data	283 (33.4)	30 (11.7)
Unreliable internet		
Respondents to question	482 (56.9)	227 (88.3)
No. (%) of respondents	144 (29.9)^c^	71 (31.3)^c^
Missing data	365 (43.1)	30 (11.7)

a The study period was January 2020–September 2022. The project was funded by the Centers for Disease and Control’s Division of Diabetes Translation DP18-1817 project, a 5-year cooperative agreement, which launched October 1, 2018, and ends September 30, 2023.

b Unless otherwise indicated, values are number (percentage).

c Percentages are based on number of respondents who answered question.

d Promise Zones are high-poverty, often medically underserved communities where the federal government partners with local leaders to enhance public health (18).

Of those who were referred by Alliance frontline staff, 257 (30%) were eligible and enrolled in an LCP. Of these, 188 enrolled in the DPP and 76 enrolled in the blood pressure self-monitoring program; 7 people enrolled in both programs. On average, those who enrolled were aged 55.3 years. Most (92%) were female, 45% lived in the Promise Zone, 14% were food insecure, 9% had transportation needs, 1% had childcare needs, and 31% had unreliable internet (Table 3).

### Focus group

Six of 15 Alliance frontline staff members participated in the focus group. Two main themes emerged from the data (Table 4). First was the importance of the frontline staff to the Alliance efforts. They described their work as “relationship-building” with patients and indicated they felt comfortable asking them about unmet social needs. They also reported serving as a resource person for many of their patients’ needs, often joining forces with each other to find resources that fit. The frontline staff noted that a main responsibility is to help patients prioritize and address stressors such as immediate obstacles and identify resources in a scarce environment. They mentioned the importance of consistent updates with patients on progress for obtaining resources, so they can move to the point where they might consider an LCP. The second theme from the focus group was barriers to patient health. The frontline staff discussed how many of their patients are focused on survival and not on healthy eating or even disease prevention. They noted that patients without basic necessities “can’t even see that as a goal,” which makes it difficult to refer them to an LCP. These barriers to patient health were amplified by the impact of the COVID-19 pandemic. The frontline staff talked about creating a place where they could share information on resources to provide to their patients and develop a cohort among themselves to “share stories and information” that might make their job easier. In the end, they reported that this could help patients be able to address their unmet needs.

**Table 4:** Themes and Example Quotes From Focus Groups With Alliance Frontline Staff in a Project to Increase Participation of Black People in Evidence-Based Lifestyle-Change Programs, St. Louis, 2018–2023^a^

Theme	Example quotes
Theme 1: Importance of frontline staff to Alliance efforts	I think one of the benefits of having community health workers screen for social determinants of health is that they are experts in developing that relationship and that rapport to be able to access information.
	It depends on that rapport that that CHW [community health worker] or CRC [community resource coordinators] or whoever originally builds with the patient. That carries a long ways. If you come off like you know everything, you will not get answers. You will get just what they want to tell you. You have to be a person to them.
	A lot of these things really affect people in ways that you might not think about unless you’re really, really working with them every day.
Theme 2: Barriers to patient health	Our patients certainly struggle with transportation, food and childcare, but to me it’s sometimes just the tip of the iceberg. There’s all of the different adverse community experiences they’ve had. Discrimination, poverty. A lot of different traumatic events that they’ve experienced. And so, then that’s just another layer we have to consider when we’re helping them to work through transportation, food, childcare and other social determinants. Because there’s always layers of social and structural determinants of health that we have to address.
	We have patients who don’t have electric or gas, they don’t have a refrigerator, they don’t have some things that some people might consider basic. That’s their starting point. So, we have to start at their starting point, which sometimes is not necessarily focusing on healthy eating. So, we try to help them get those needs met so we can get them to a starting point of focusing on health.

a The study period was January 2020–September 2022. The project was funded by the Centers for Disease and Control’s Division of Diabetes Translation DP18-1817 project, a 5-year cooperative agreement, which launched October 1, 2018, and ends September 30, 2023.

### Training center

In year 3, a total of 13 frontline staff members participated in trainings offered by the training center (Table 5). Of the 13 participants, 6 worked for the Missouri Primary Care Association, 2 worked for the Integrated Health Network, 1 worked for the St. Louis County Department of Public Health, 2 worked for the Gateway Regional YMCA, and 2 worked for the City of St. Louis Department of Health. On average, trainees completed 4.2 training modules during year 1. Of the training modules offered in year 3, three addressed health equity, 1 addressed trauma-informed care, 2 addressed mental health, 3 addressed health literacy, and 3 addressed racial equity.

**Table 5:** Summary of Trainings Completed, by Domain, in a Project to Increase Participation of Black People in Evidence-Based Lifestyle-Change Programs, St. Louis, 2018–2023^a^

Training name	Domain (no. of modules)					No. of participants per training module
	Health equity (n = 3	Trauma-informed care (n = 1)	Mental health (n = 2)	Health literacy (n = 3)	Racial equity (n = 3)	
Unequal Treatment: Disparities in Access, Quality, and Care	X				X	7
No Safety, No Health: A Conversation about Race, Place and Preventing Violence		X			X	8
Let’s Live Healthy! High Blood Pressure in Pregnancy				X		5
Mental Health and Wellness: Positive Psychology and Psychiatry in Uncertain Times			X			9
Understanding Health Disparities in Heart Disease in these Unsettling Times	X				X	7
The Importance of Measuring Blood Pressure Accurately				X		4
Understanding the Intersection of Diabetes and Addiction	X		X			7
Use of Social Media and Peer Support in Diabetes Care: A Panel from AADE Project Leaders				X		7

Abbreviation: AADE, Association of Diabetes Care & Education Specialists.

a The study period was January 2020–September 2022. The project was funded by the Centers for Disease and Control’s Division of Diabetes Translation DP18-1817 project, a 5-year cooperative agreement, which launched October 1, 2018, and ends September 30, 2023.

Trainees reported gaining several skills from the modules, including the ability to understand their role in the Alliance and monitor personal bias. Trainees also developed interpersonal and professional skills, including de-escalating crisis situations, fulfilling mandates for reporting, educating patients on mental health, and monitoring patients’ exercise and health. Lastly, trainees developed skills to understand the influence of community and environmental factors on health equity. When asked how these skills would affect their ability to refer patients, trainees reflected on asking appropriate questions, understanding correct procedures, communicating their role to patients, and referring patients to appropriate LCPs and community resources. One trainee commented that the training modules helped them engage with patients in an “unconventional” way by considering their “interests, values, and culture.”

## Implications for Public Health

Lessons learned from implementation and evaluation can inform other complex partnerships between clinical and community-based organizations to reduce barriers stemming from interpersonal and structural racism and increase enrollment and retention in LCPs of people disproportionately affected by chronic diseases. This 5-year real-world intervention has several public health implications. Enrolling and retaining Black people in community- and evidence-based LCPs can reduce the unequal burden of chronic disease (24). The project provided an opportunity to document evaluation and implementation facilitators and barriers that may apply to future public health efforts. We have summarized lessons learned and potential strategies for improvement.

### Understanding context and complexity

The Alliance is a partnership of multiple health organizations with various structures, systems, cultures, and priorities. Implementation science frameworks such as the Consolidated Framework for Implementation Research (CFIR) illustrate the multilevel factors within and outside an organization that affect implementation (25). The Alliance used an intentional, participatory implementation and evaluation planning approach to understand each partner’s current systems and ensure that the intervention and evaluation fit the context of each organization. This fit also included gaining an understanding of each organization’s workflow and employee responsibilities. The evaluation was planned in collaboration with our partners to leverage existing data and expand their capacity for systematic and rigorous data collection. Each organization had multiple people in 2 key roles for implementation: managers and frontline staff. Developing communication structures that ensured all implementers and evaluators had a common understanding of the Alliance goals, implementation processes, and requirements for data reporting was critical. For example, frontline staff members were encouraged to provide feedback immediately after each training module, which helped the project manager and evaluators amend topics and modalities for subsequent training modules and evaluations. Compounding the implementation and evaluation was the evolution of systems, processes, priorities, and people throughout the project period, which likely was heightened by the COVID-19 pandemic. Changes in data collection methods and platforms affected data consistency and quality (eg, pre- and postsurvey data from the training center were not usable). Furthermore, COVID-19 placed unforeseen demands on Alliance partners that left staff stretched thin and unable to fully complete the planned project and evaluation activities within the intended time frame.

When working with racial and ethnic minority populations who are potential participants in LCPs, it is also critical to understand the context (eg, environments) and complexity (eg, life situations, competing demands, diverse needs) of their lived experience that translate into barriers to meeting their needs. Our frontline workers were valued members of the community; they understood and established trust in the community. Having nonjudgmental, truthful conversations about social needs allowed for meaningful intervention. On the other hand, the context of each encounter (eg, limited time, lack of privacy) was not always suitable for certain conversations or referral to an LCP.

### Developing collective, multilevel buy-in and prioritization

Partnerships between community- and clinic-based organizations and researchers offer an opportunity to bring scientific and practice-based knowledge and experience together to improve the quality, value, and relevance of implementing interventions. To achieve meaningful public health impact, a diverse set of clinical and community programs and partners is needed (26). Residents must use multiple assistance and intervention resources to ensure their needs are met (27). To this end, the Alliance comprises various organizations (eg, clinics, health departments, community-based organizations, universities) and multiple partners with various roles (eg, implementers, managers, evaluators, funders). The effective delivery of interventions requires engagement and buy-in at multiple levels. The field of implementation science has emerged as a response to the challenges in translating evidence-based practices to real-world settings (28,29). Attention is paid to pre-implementation, which is the work necessary to effectively engage organizations and staff. Co-development of project goals, particularly with frontline staff, from inception may have generated stronger commitment and understanding of Alliance goals. Furthermore, clearly communicating implementation and evaluation expectations for each partner is vital to success. One facilitator of the Alliance’s success in generating buy-in was the quarterly data report, which was disseminated via email and a shared drive and presented in all-partner meetings. These reports allowed partners to review collective progress and how this progress contributed to common goals. Additionally, the bootcamp-style training helped communicate project goals and structure to new Alliance members. Our intention was not to rigorously study these strategies; however, such a study could contribute to the field of implementation science by expanding the understanding of the mechanisms of change and the effectiveness of these discrete, multifaceted, and tailored strategies (30).

### Being flexible and adapting

The Alliance evolved and responded to consequences of the COVID-19 pandemic in both engagement and service delivery. The COVID-19 pandemic started in year 2 (October 2019–September 2020) of this project, causing major shifts in priorities and resources as partners re-allocated staff to respond. Despite these shifts, engagement and enrollment in our programs increased, albeit slightly, each year. Although the main goal of the Alliance was maintained throughout the pandemic, flexibility was needed not only from partners but also from project funders, evaluators, and leadership. Some planned activities were delayed, while others sped up to support the community during the public health crisis. For example, an original program goal was to develop an online telehealth platform for DPP participants in year 4 (October 2021–September 2022). This goal was expedited. In year 3, we offered new remote classes, such as a lunchtime 30-minute exercise class and FitBit challenges, to all LCP enrollees. In addition to an online DPP course that was delivered by lifestyle coaches in a synchronous format, the Alliance piloted a self-paced online DPP program for 22 people. As a result of the effectiveness and acceptability among pilot participants, the Alliance opened referrals to anyone interested in this program. The community members’ feedback was invaluable in developing this program.

Virtual LCPs became the only option for participating in an LCP during the COVID-19 pandemic. Virtual classes can improve access for people with transportation or time barriers or limited access to technology devices or reliable broadband internet. Frontline staff were primed with resources (eg, the Affordable Connectivity Program offered by the Federal Communication Commission, library hotspots) to support people without internet access or in places with poor connectivity. Enrollees were further supported by lifestyle coaches. Infrastructure changes and additional resources are needed to fully support these people and improve digital literacy among populations who may not be comfortable using technology (eg, older persons).

Another example of the impact of the COVID-19 pandemic was flexibility in recruitment methods. Before the pandemic, community members were encountered primarily through in-person clinic visits, community events, and health fairs. During the pandemic, the Alliance shifted strategies to reach people remotely (eg, via telehealth, telephone) and launched a marketing campaign that promoted LCPs at transit stops and via social media. The Alliance leveraged increases in drive-through food distributions by including flyers about the Alliance program and the DPP in food boxes. The Alliance also increased community awareness of food resources by building a website that provides details of mobile grocery vendors and other food access opportunities.

Another adaptation to the COVID-19 pandemic was to change frontline staff trainings to a flexible, self-paced format and add COVID-19–related material (eg, a training titled “Understanding Health Disparities in Heart Disease in these Unsettling Times”). The Alliance also pivoted to support the needs of communities and partners. For example, frontline staff in clinical settings received training in a COVID-19 vaccine module to assist community members who were not vaccinated and had questions about the vaccine. To maintain project goals, vaccine appointments were leveraged as an opportunity to screen and assist with unmet social needs, particularly because these needs had increased during the pandemic among racial and ethnic minority groups.

Evaluating a constantly adapting project was a challenge. These adaptations required bidirectional communication with implementers and project managers to ensure progress toward intended goals. Annual documentation of progress was also required by the funder. Collaborative relationships between the Alliance evaluation team and partners were key to overcoming this challenge.

### Keeping an eye to the future

To fully realize public health impact, we should broadly and equitably sustain effective public health programs and partnerships; this sustainment requires active and early planning (31). The Alliance evaluation will use a participatory design approach for developing a sustainability plan and generating capacity for sustainability. Sustainability capacity, defined as the ability to maintain systems and their benefits over time, may be influenced by 8 domains outlined in the sustainability framework: environmental support, funding stability, partnerships, organizational capacity, program evaluation, program adaptation, communications, and environmental support (32,33). To build capacity, it is necessary to systematically assess and understand factors affecting a program’s sustainability capacity and develop a sustainability plan with actionable strategies. The Alliance will use a mixed-methods, partner-engaged approach involving quantitative surveys and qualitative interviews. We first want to understand perceived barriers (eg, resources, time) and facilitators within these 8 domains to continue the Alliance partnership and referral system. The use of such an approach to ensuring sustainability is essential to public health impact and is required by many public health agencies and foundations (eg, CDC, Robert Wood Johnson Foundation, Kaiser Permanente).

### Conclusion

Responding to complex health inequities in communities requires collaborative partnerships. The Alliance is an example of how health care, public health, and community partners work together to increase recruitment and enrollment of racial and ethnic minority populations who are disproportionately affected by chronic diseases into evidence-based LCPs. Solely increasing access to these programs may not achieve the desired effect. The Alliance also aims to address interpersonal and structural racism that may generates barriers (eg, structural barriers to food access, physical activity facilities, childcare, and transportation) that impede equitable health improvements. The Alliance evaluation shows that strong collaborative relationships among partners and the co-development of systems and priorities can achieve positive outcomes.
